# Two-Dimensional Manipulation in Mid-Air Using a Single Transducer Acoustic Levitator

**DOI:** 10.3390/mi10040257

**Published:** 2019-04-18

**Authors:** Harri Wijaya, Kourosh Latifi, Quan Zhou

**Affiliations:** Department of Electrical Engineering and Automation, Aalto University, 02150 Espoo, Finland; harri.wijaya@aalto.fi (H.W.); kourosh.latifi@aalto.fi (K.L.)

**Keywords:** acoustic levitation, contactless manipulation, Chladni plate

## Abstract

We report a single transducer acoustic levitator capable of manipulating objects in two-dimensions. The levitator consists of a centrally actuated vibrating plate and a flat reflector. We show that the levitation position of the object depends not only on the vibration frequency, but also on the tilting angle between the plate and the reflector. Additionally, new levitation positions can be created by actuating the plate with a composite signal of two frequencies using frequency switching. Based on recorded levitation positions, such single transducer acoustic levitator can manipulate a cluster of levitated microspheres in predefined trajectories, with mean position error of 155 ± 84 µm.

## 1. Introduction

Acoustic manipulation is a material-independent method having a broad range of biomedical and material applications [1,2,3,4]. Acoustic manipulation can be achieved using techniques such as contact force [5,6,7], streaming [8,9,10], and radiation force [11,12,13]. The latter gives rise to acoustic levitation that can suspend objects in the mid-air and has attracted wide interests. A typical acoustic levitator consists of an emitting surface and a reflector or a pair of opposing emitting surfaces in order to form a pressure standing wave. A levitator with a single-sided emitter has also been developed [14,15]. The primary acoustic radiation force, arising from the nonlinear property of the acoustic pressure field, will move an object heavier than air toward the pressure node. If the force is strong enough to overcome the gravitational force, the object will be levitated and trapped around the pressure node, also referred to as the acoustic trap.

Acoustic levitation employs either a static-field or a dynamic-field (see a recent review by Drinkwater [16]). Static-field levitation can suspend objects in fixed locations [2,17], while dynamic-field levitation allows contactless transport in one [18,19,20], two [13,21], and three dimensions [14,22,23]. Contactless transport by means of acoustic levitation can be achieved in various ways. Bjelobrk [18] employed a single transducer and controlled the location pressure node by varying the distance between emitting surface and reflector. Koyama [19], Thomas [20], and Kashima [21], in their independent setups, varied the flexural mode of the vibrating plate by controlling the phase difference of the two transducers or the two pairs of transducers. Franklin [23] used a 3D-printed Fresnel lens and a two-element disk to produce an acoustic trap, and employed a translation stage to move the whole transducer system and accordingly the acoustic trap. Foresti [13] used an array of transducers under a reflector and applied spatiotemporal amplitude modulation on the transducer array to create an acoustic trap at a desired position. Ochiai [22] used opposing ultrasonic phased arrays to generate a pressure node at an arbitrary position, and moved it in 3D by reprogramming the time delay in each individual transducer. Marzo [14] introduced the holographic acoustic elements framework for an ultrasonic phased array that allowed the generation of multiple acoustic traps in the positions according to the holographic focusing element, even with a single-sided array. In summary, the dexterity of dynamic-field levitation requires an extra manipulation tool [18,23] or multiple individually-controlled transducers [13,14,19,20,21,22].

In this paper, we report a simple acoustic levitator that can manipulate objects in two dimensions using just a single transducer and a reflector, without any moving element except the transducer. We firstly identified the levitation position (or acoustic trap) for different actuation signal frequencies. Then, we adopted a frequency-switching method to generate new levitation positions. Finally, we demonstrated the capability of our single transducer levitator to move an object following 2D trajectories. To the best of our knowledge, this is the first work showing a single transducer acoustic levitator able to manipulate an object in 2D trajectory by solely controlling the actuation signal.

## 2. Methods

### 2.1. Experimental Setup and Material

**Figure 1:**
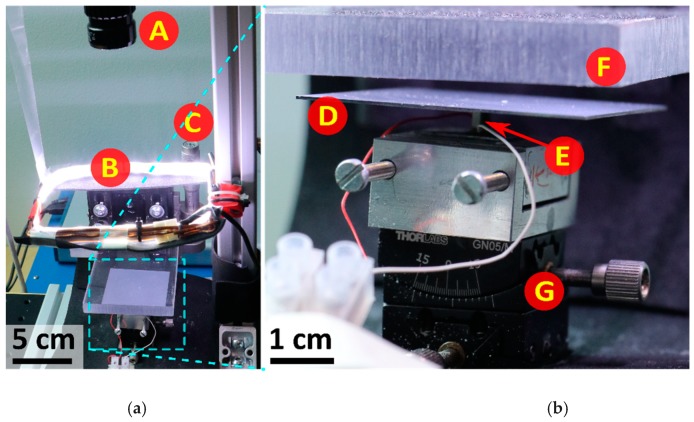
Photograph of the experimental setup (**a**) and closer view of the main components (**b**). A—camera and video lens, B—light-emitting diode (LED)-illumination, C—translation stage with micrometer, D—plate, E—piezoelectric stack actuator, F—reflector, G—dual-axis goniometer.

The acoustic levitator experiment setup is shown in Figure 1. The main components were a vibrating plate as the transducer and a flat reflector. The plate (silicon, 50 mm × 50 mm × 525 μm) was centrally-actuated to generate the flexural vibration mode on its surface. The actuator was a piezoelectric stack actuator (Pst150/2x3/5, Piezomechanik GmbH, Munich, Germany) and was glued using cyanoacrylate adhesive on to center of the surface of plate. The actuator was driven by a signal composed in a PC using MATLAB (MathWorks, Natick, MA, USA), converted to analog signal by a digital-to-analog board (NI USB-6341, National Instruments, Austin, TX, USA), and amplified by a linear amplifier (EPA-104, Piezo Systems, Woburn, MA, USA). A flat transparent reflector (polycarbonate, 10 mm thickness) was mounted above the transducer. The vertical position of the reflector was adjustable using a manual linear stage (M-423, Newport, Irvine, CA, USA). The plate and the actuator were mounted on a dual-axis goniometer (GN2/M, Thorlabs, Newton, NJ, USA) to set the tilting angle between plate and reflector. The levitated object and plate were imaged by a camera (IGV-B1621C-KC000, ImperX, Boca Raton, FL, USA, with Infinity/InfiniMite Alpha lens) mounted above the plate and reflector.

The levitated objects in this paper were a cluster of microspheres (092DET100d25, AkzoNobel, Amsterdam, The Netherlands), where the size of each individual microsphere was 80–120 µm and the density was 25 ± 3 kg/m^3^. The size of the cluster in our experiment was about 1 mm.

### 2.2. Manipulation Method

**Figure 2:**
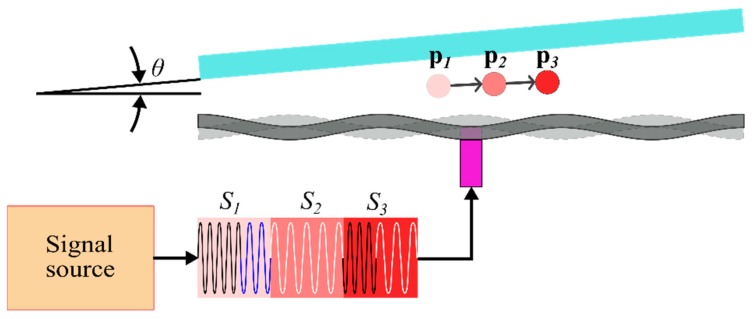
Schematic of the acoustic levitator; a particle is levitated at position $p_{i}$ when signal $S_{i}$ is played, i = 1,2,3. Tilting angle between surfaces of the transducer and the reflector is θ.

We propose a non-contact method for an acoustic levitator to manipulate the levitated object between two non-parallel surfaces, the vibrating plate and reflector, by controlling only the plate vibration frequency. A simplified schematic of the acoustic levitator, which also serve as the concept of our proposed method, is shown in Figure 2.

For acoustic levitator in air, the dominant acting forces are gravitational force and acoustic radiation force. If acoustic radiation force is sufficient to overcome the gravitational force, hence the total acting forces is zero, the object is then levitated and trapped at a point in mid-air. We refer to such point as levitation position in the rest of this paper. The total force acting on an object can be calculated as:

(1) $$F_{total}=-\nabla U_{total}=-\nabla \left(U_{grav}+U_{aco}\right)$$

where $U_{grav}$ and $U_{aco}$ are gravitational potential and acoustic potential (or Gorkov potential), respectively. The levitation position, where total force is zero, is identified by the local minima of the total potential. The gravitational potential can be calculated as:

(2) $$U_{grav}=\rho_{p}V_{p}gy$$

where $\rho_{p}$ and $V_{p}$ are density and volume of the object, g is gravitational acceleration, and y is vertical coordinate in the direction of gravitation. The acoustic potential can be calculated as:

(3) $$U_{aco}=V_{p}\left(f_{1}\frac{1}{2}\kappa_{0}\langle {\left|p_{1}\right|}^{2}\rangle -f_{2}\frac{3}{4}\rho_{0}\langle {\left|v_{1}\right|}^{2}\rangle \right)$$

where $\langle {\left|p_{1}\right|}^{2}\rangle$ and $\langle {\left|v_{1}\right|}^{2}\rangle$ are the mean squared of first order pressure and first order particle velocity at the object, respectively. $v_{1}$ can be calculated from $p_{1}$ as $v_{1}=-\nabla p_{1}/j\omega \rho_{0}$, where ω is frequency of the sound pressure and $\rho_{0}$ is density of medium (in this case is air). $f_{1}$ and $f_{2}$ are dimensionless scattering coefficients that can be calculated as $f_{1}=1-\frac{\kappa_{p}}{\kappa_{0}}$ and $f_{2}=\frac{2\left(\rho_{p}/\rho_{0}-1\right)}{2\rho_{p}/\rho_{0}+1}$, where κ and ρ are compressibility and density, respectively, whereby subscript p denotes the object and 0 denotes the medium.

**Figure 3:**
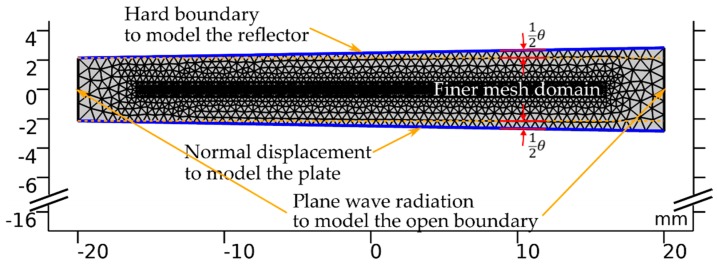
Geometry, mesh, and boundary condition for finite element analysis of plate-reflector model.

The manipulation principle of the proposed method is based on the displacement of the levitation positions at different actuation frequencies in a nonparallel plate-reflector system. In order to study the contribution of the plate-reflector tilting angle and the vibration frequency to the levitation position, we performed a finite element analysis (FEA) of the plate-reflector model in COMSOL Multiphysics (version 5.4, Stockholm, Sweden) to calculate the total potential over the entire region between the plate and the reflector, and then identified the levitation position. Figure 3 shows the geometry, the boundary conditions, and the mesh of the model. To achieve high spatial resolution, we set the mesh to be finer in the center region, where the levitation position is expected to occur.

**Figure 4:**
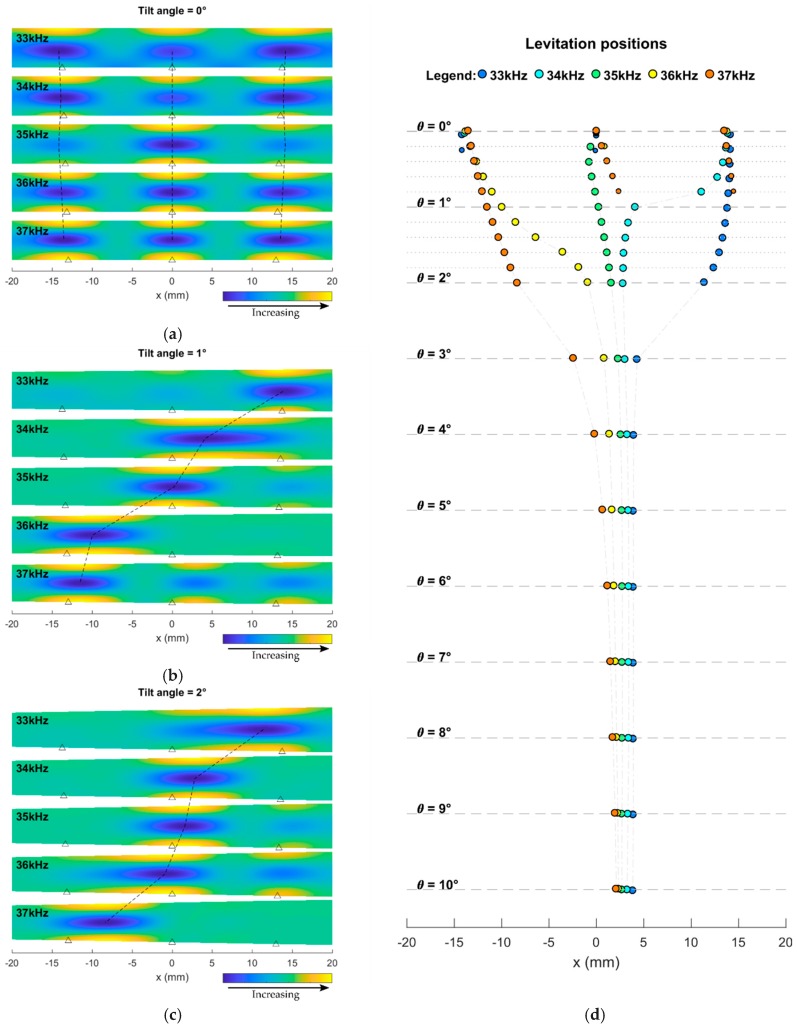
Normalized potential at the frequencies of 33–37 kHz for three different tilting angles: (**a**) 0°, (**b**) 1°, and (**c**) 2°. Triangle symbols represent the flexural antinodes. Dashed lines guide the eye. (**d**) Levitation positions for the tilting angles of 0.0°–1.8° with step of 0.2° and the tilting angles of 2°–10° with step of 1°. Marker radius is inversely proportional to the potential value.

Figure 4a–c show the normalized potential for the tilting angles of 0°, 1°, and 2°, at multiple frequencies (33–37 kHz). Figure 4d shows the simulated levitation positions for multiple tilting angles (0°–10°) and frequencies (33–37 kHz). Our simulations show that at the tilting angles of 0°, multiple levitation positions occur for each frequency. However, there is little difference between the levitation positions of different frequencies. By increasing the tilting angle to around 1°–2°, the levitation positions of different frequencies diverge, allowing position control using actuation frequency. Nevertheless, further increase of the tilting angle converges the levitation positions.

Figure 4 also shows that the displacement of levitation position against the actuation frequency is not monotonic. For example, at tilting angle of 1° (Figure 4b), changing frequency from 33 to 34 kHz leads to about 10 mm displacement leftwards, where the plate-reflector distance is smaller; the value is about 4 mm from 34 to 35 kHz; and about 10 mm displacement from 35 to 36 kHz.

## 3. Results and Discussion

### 3.1. Maps of Levitation Position versus Vibrating Frequency

**Figure 5:**
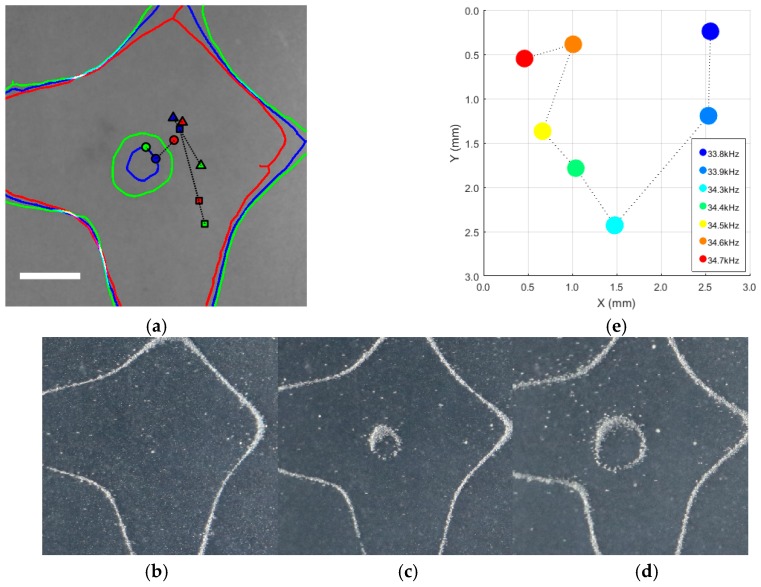
(**a**) Levitation position of the particle at three vibration frequencies (red: 33.8 kHz, blue: 34.4 kHz, green: 34.8 kHz) for three tilting angles (□: 0°50′, Δ: 1°0′, ○: 2°0′); 25 × 25 mm center crop of the actual plate. The solid lines are the nodal lines of the flexural vibration mode of the plate for the three frequencies. Nodal lines in (**a**) are extracted from (**b**–**d**) Chladni figures on the vibrating plate with vibration frequencies of 33.8, 34.4, and 34.8 kHz, respectively. (**e**) Levitation positions driven by single frequency. Position (0.0, 0.0 mm) corresponds to position (24.0, 23.5 mm) in the plate coordinate.

We employed a square plate and a reflector to simultaneously levitate and manipulate particles in ambient air environment following a 2D trajectory. We spread the microspheres, as levitated objects, on the top of the surface of the plate. The plate was then actuated to generate standing sound waves between plate-reflector, and eventually levitate and trap the microspheres. The microspheres self-assembled and formed a cluster, about 1 mm in size. We examined multiple tilting angles, and observed that the three angles, 0°50′, 1°0′, and 2°0′, provided significant spread of particle positions when the frequency varied. Figure 5a shows the levitation positions of a cluster of microspheres at three frequencies and three tilting angles. Nodal lines were obtained by processing the images of Chladni figures, Figure 5b–d, from the sand grains experiment using the morphological shrink algorithm. It is apparent that the levitation position do not necessarily displace monotonically in the *x*-direction, nor in the *y*-direction, as the frequency increased. The levitation position to frequency relation are to be obtained experimentally.

Among the three angles, the angle 2°0′ provided a spread that minimized the difference in Euclidean distance between each pair of particle positions, or maximized the spread area. In the following study, we fixed the tilting angle at 2°0′. The distance between the center of the plate and the reflector surface was 5.4 mm, which corresponds to about a half-wavelength of the sound generated at the excitation frequency. As the levitator creates a rather complicated acoustic field across the plate, we focused our study to the 3 mm × 3 mm region of interest around the center of the plate in this paper.

The relation between levitation positions and frequencies is shown in Figure 5e. We scanned the frequency from 33.8 to 34.7 kHz, with a step size of 0.1 kHz. We provided a fixed signal amplitude of 24 V except for 33.9 kHz, for which we provided 30 V to improve levitation stability. Seven frequencies, namely 33.8, 33.9, 34.3, 34.4, 34.5, 34.6, and 34.7 kHz, led to stable levitation positions, while other frequencies did not lead to stable levitation and were discarded. For the seven frequencies, the levitation positions corresponded to the frequencies spread within the region of interest. However, the connecting lines between adjacent levitation positions on all levitation positions formed a U-shaped curve. Therefore, it was challenging to obtain levitation positions beyond the curve by using finer steps of frequencies (e.g., within the area enclosed by the U-shaped curve).

### 3.2. Establish New Levitation Positions by Frequency-Switching

**Figure 6:**
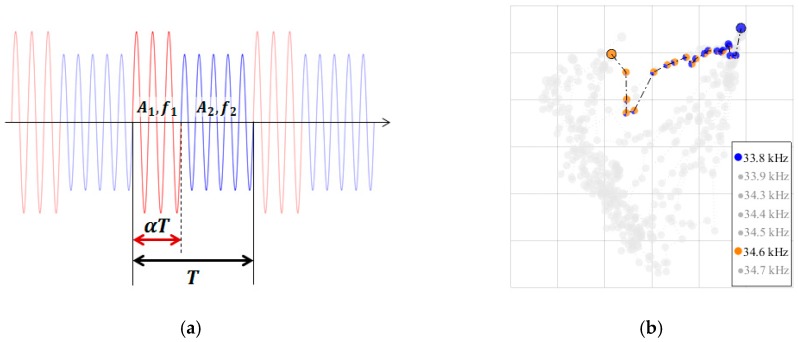
(**a**) Illustration of composite signal. The signal consists of a sequence of chunks with duration *T*. Each chunk consists of first signal (amplitude *A*_1_, frequency *f*_1_) with duration of *αT* followed by second signal (amplitude *A*_2_, frequency *f*_2_) of duration (1-*α*)*T*. (**b**) New levitation positions created by two composite signals (33.8 and 34.6 kHz). The area of certain color (correspond to one frequency) in each pie-like marker is proportional to the duration ratio of the corresponding frequency.

To increase the reachable area, we applied a frequency-switching method, inspired from the work of Glynne-Jones [24] for one-dimensional manipulation. The frequency-switching method employs a composite signal y(t) that can be described by:

(4) $$y\left(t\right)=\{\begin{array}{cc} s\left(A_{1},\;f_{1},\;t\right), & t\;\mathrm{mod}\;T\leq \alpha T\\ s\left(A_{2},f_{2},t\right), & t\;\mathrm{mod}\;T>\alpha T \end{array}$$

where s is a sinusoidal signal of amplitude A and frequency f, t is time, T is the period of composite signal, and α∈[0,1] denotes the duration ratio between the duration of the first signal and T. See Figure 6a for the illustration of this signal composition. The period of composite signal T is given by [24]:

(5) $$T\ll \frac{3\mu rc}{2fA_{f}}$$

where μ is the dynamic viscosity of medium, r is the radius of the object, c is the speed of sound in the medium, and $A_{f}$ is the amplitude of force profile. As approximation, by using the weight of single particle as the amplitude of force profile yield T≪270 ms for frequency of 34.7 kHz. The period of composite signal T=10 ms was used in our experiment.

**Figure 7:**
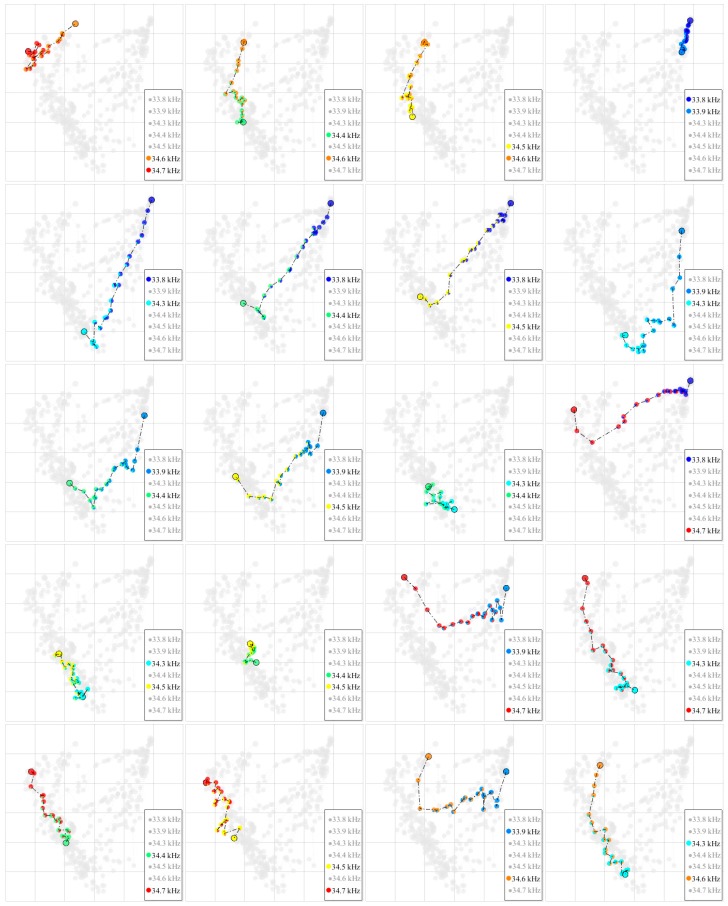
Levitation positions of composite signals for the remaining 20 pairs of combinations from the seven frequencies, excluding one combination of 33.8 and 34.6 kHz.

Figure 6b shows the levitation positions of two composite signals (33.8 and 34.6 kHz), with α varying from 0 to 1 in a step of 0.05. Noticeably, the levitation positions do not fall on the line segment between levitation positions of two single frequency signals. We attribute this non-linearity to the complex flexural vibration mode of the plate. We conducted experiments to record the levitation positions of composite signals for all 21 pairs of combinations from the seven frequencies. The results of the remaining 20 pairs are shown in Figure 7.

### 3.3. Trajectory Following Demonstration

**Figure 8:**
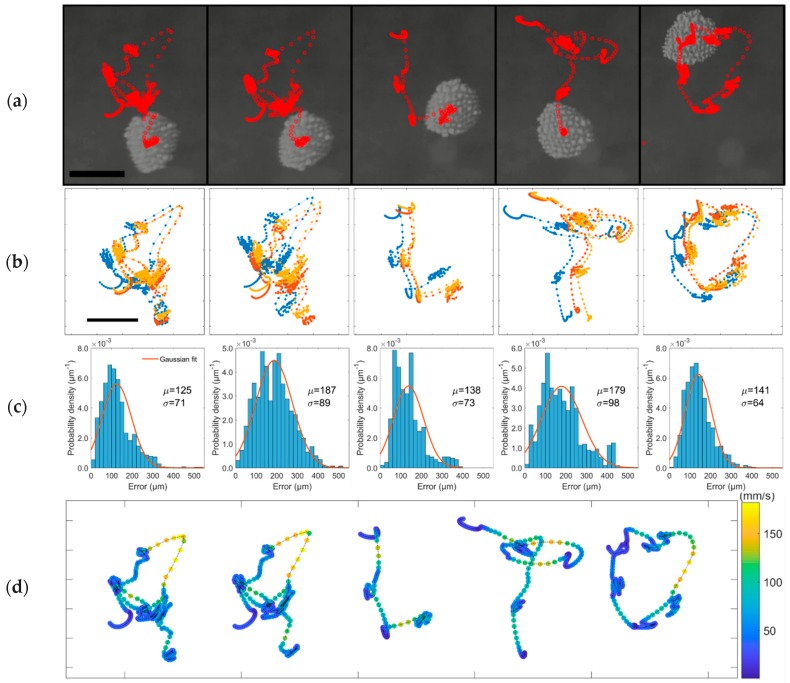
(**a**) Trajectory following results forming the letters AALTO. The red circles indicate the position of the microspheres cluster at each video frame (frame rate is 1000 FPS). (**b**) Trails for three repetitions of the trajectory following the experiment. (**c**) Position error histogram and corresponding Gaussian fit; the error is measured as the deviation from the mean trajectory of all repetitions. (**d**) The distribution of speed of the cluster on the mean trajectories. The scale bar is 1 mm in (**a**,**b**).

Using the saved composite signal and position relation, we can levitate and manipulate a cluster of microspheres in predefined trajectories. We first designed a trajectory consisting of waypoints, then matched each waypoint to the closest levitation position we had collected, resulting in a sequence of composite signals. We then played the sequence of signals to levitate and move the microspheres to follow the designed trajectory in an open-loop control. In Figure 8a, we demonstrate five sequential trajectories that resemble letters AALTO, where the time duration of each step is 50 ms. The motion of the self-assembled cluster of microspheres was recorded using a high-speed video camera (Phantom v2012, Wayne, NJ, USA) with a macro lens (Canon MP-E 65 mm f/2.8 1-5X Macro Photo, Tokyo, Japan) at the frame rate of 1000 FPS. The focal plane is set to be between the plate and reflector. Since the cluster is self-assembled, the cluster adapts its size to the levitation positions where some particles would leave the cluster if the size of a levitation position is smaller than the size of the cluster (see Supplementary Video S1).

We repeated the experiment three times and quantified the repeatability by calculating the position error as deviation from the mean trajectory for all repetitions. Figure 8b shows the trajectories in all repetitions and Figure 8c shows the corresponding position error histogram. The mean value of all position errors was 155 ± 84 µm. The side length of the trajectory was about 2 mm, hence the mean position error was about 7% of the trajectory side length. We attribute the spatial shift in the trajectories (Figure 8b) to temperature variation. The temperature variation changes the speed and wavelength of sound, leading to the spatial shift of the levitation position. We also calculated the speed of the cluster for each trajectory in all repetitions, where the speed is calculated over three consecutive frames. Figure 8d shows the distribution of the speed plotted on mean trajectories. It is apparent that the cluster moves between levitation positions at a higher speed and settles around the levitation position at a lower speed, and the maximum speed is 182 mm/s.

## 4. Conclusions

In summary, we have demonstrated a simple single-transducer acoustic levitator capable of manipulating objects in a two-dimensional trajectory. The levitator utilizes the feature that the levitation position changes at different vibration frequencies when the plate and the reflector have a small angle. The spread of the levitation positions depends on the tilting angle between the plate and the reflector. Tilting angle is chosen to balance between the spread of levitation positions and the one-to-one mapping of frequency to distinct levitation positions. The reachable area can be increased by composite signals of two frequencies using frequency-switching.

In the demonstration experiment, the levitated object follows the 2D predefined trajectories. The object reaches a speed up to 182 mm/s and the average deviation from mean trajectory is 155 ± 84 µm. It is possible to reduce the deviation by using a controlled environment to minimize air temperature variation.

Our method opens the path to a new design of acoustic levitation systems. Unlike other acoustic levitators that require multiple independently-controlled transducers to achieve 2D manipulation, our method is extremely simple requiring only a single transducer. Despite its simplicity, it allows manipulation of a cluster of micro objects in predefined 2D trajectories. We envision that the proposed method has great potential for simplification of micro-manipulation of levitated objects. This can benefit numerous applications in micro-assembly [11,25], material characterization [26,27], and transport and diagnosis of biological samples [28,29,30].

## Supplementary Materials

The following is available online at https://www.mdpi.com/2072-666X/10/4/257/s1, Video S1: Trajectories resemble letters AALTO.
